#  Subjective Visual Vertical and Postural Performance in Healthy Children 

**DOI:** 10.1371/journal.pone.0079623

**Published:** 2013-11-13

**Authors:** Chrystal Gaertner, Maria Pia Bucci, Rima Obeid, Sylvette Wiener-Vacher

**Affiliations:** 1 Université Pierre et Marie Curie, Paris, France; 2 Children balance Evaluation Center, Robert Debré Paediatric Hospital, Paris, France; 3 UMR 676, INSERM, Université Paris 7, Hôpital Robert Debré, Paris, France; Tokai University, Japan

## Abstract

**Introduction:**

Verticality is essential in our life, especially for postural stability. Subjective vertical as well as postural stability depends on different sensorial information: visual, vestibular and somesthesic. They help to build the spatial referentials and create a central representation of verticality. Children are more visuo-dependant than adults; however, we did not find any study focusing on how children develop their sense of verticality.

**Methods:**

We studied two groups of subjects: 10 children (from 6 to 8 years) and 12 young adults. We recorded postural stability with a Techno Concept plateform and perception of subjective visual vertical in the following conditions: while adjusting the vertical in the dark or with visual perturbation, while fixating the vertical bar, and with eyes closed.

**Results:**

Children are more instable than adults in terms of postural parameters, and also while performing a double task, especially when no visual references are present. They also present a higher variability and lower accuracy than adults in reporting their perception of true vertical reference.

**Discussion:**

Children might have limited attentional resources, and focus their attention on the more demanding task, corresponding to the U-shaped non-linear model.

## Introduction

The verticality is essential in our life; it is a perception of gravity built shaped from three sensory modalities. It is essential for bipedal orthostatic posture imposing a very small contact area to the ground in reference to body height, source of instability. The various motor behaviours organized around the vertical axes such as body orientation and stabilisation in space as well as locomotion and spatial navigation [1] are dependent upon both perception and integration of verticality. Humans, assess an object verticality through the perception of its image on the retina, which is relative to the eye position and the head direction [2]. Consequently, the reference to visual verticality may sometimes be missing in our environment. For this reason an internal model of verticality is essential to allow development of postural stability and body control in the environment [3,4]. The subjective visual vertical, as well as the postural activity, are built from integration of different sensorial information: visual, vestibular and somesthesics [5].

Mittlestaedt [2] proposed a three-vector computational model to be used to determine subjective visual verticality. According to this model, the visual and gravitational vectors localize the physical zenith, while the idiopathic vector of central origin localizes the longitudinal axis of a person. This model has evolved to a model where the central nervous system uses three spatial different reference frames based on different information: an *egocentric reference*, where the identification of the object position is based on the subject body which depends on somesthesic information; an *allocentric reference*, where the object is localized through its spatial configuration regardless of the subject position, which depends on visual information and a *gravitational reference* which is linked to the orientation and the intensity of the gravitational vector depending on vestibular information. This third reference frame is certainly the absolute reference for the central nervous system as it is independent from both object and subject body positions [1,6,7]. All these three spatial references frames are used to create a central representation of verticality that also provides a referent vertical axis around which motor and oculomotor behavior may be organized. 

In healthy adults the presentation of a tilted visual reference frame (static visual information) induces a deviation of the body position [8]. It also results in a tilt of the subjective visual vertical [9,10] in the same direction than the tilt of the visual reference frame, despite the visual dependency of some subjects. Dynamic visual information also influences subjective visual vertical. For instance, a visual scene rotating around the eyes axis in the frontal plane creates an optokinetic stimulation and induces a tilt of the subjective visual vertical in the direction of the visual rotation [10–15]. 

To the best of our knowledge, no studies have been completed so far on the development of verticality perception in children, nor on the influences of visual information on the verticality perception and postural control in children. This study aims to define the role of visual information on the development of verticality and postural stability in children from 6 to 8 years of age. 

Lee and Aronson [16] first showed the importance of vision for standing and for postural control in infants. They studied the standing posture of seven young infants from 13 to 16 months, with optic flow pattern stimuli. They reported the backward or forward body sways of the infant, indicating compensatory adjustments to posture made in response to the visual proprioceptive information received. They found also that in the majority of cases, infants produced a sway in the same direction as the optic flow, thus making compensatory adjustments of posture according to their visual informations. These authors concluded that for control of postural stability, infants used more heavily “visual proprioception” than mechanicals proprioception informations. Visual inputs would be used more than other inputs to control posture. 

According to Assaiante [17], the construction of the spatial representation of the body and of the environment is based on perceptive references, such as verticality, which is provided by vestibular and somesthesic information based on the vertical axis or on visual information for verticality. This information is added to this reference and integrated to constitute a multi-sensorial frame of reference independent from the body. Using this knowledge, we hypothesize that children will be more instable than adults in terms of postural stability, especially in the visual perturbed environment, and will have less precise and less accurate representations of verticality than adults. This study aims to provide a better understanding of spatial perception mechanisms and of their role in the development of verticality and postural stability during childhood. 

## Methods

### Subjects

Ten children from 6 to 8 years of age (mean age 6.86 ± 0.61 years) and twelve adults from 19.8 to 27 years (mean age 22.88 ± 2.44 years) participated in this study. For these subjects, 3/12 adults were left handed, 9/12 were right handed and 8/10 children were right handed, 2/10 were left handed. All subjects had normal or corrected vision and wore their glasses during the test. All subjects underwent a complete paediatric vestibular evaluation including head impulse test, caloric test, EVAR tests for canal function, OVAR test and bon conduction VEMP for otholith function assessment, as well as a neurological and audiological evaluation [18,19]. They did not present any vestibular, ocular nor any neurologic pathology. The investigation adhered to the principles of the Declaration of Helsinki and was approved by our institutional Human Experimentation Committee (Comité de Protection des Personnes CPP Ile de France V, Hôpital Saint-Antoine). Informed written consent was obtained for each adult subject and from the children’s parents after careful review of the experimentation with the participants. All subjects were naïve regarding the purpose of the study. 

### Experimental device

**Figure 1:**
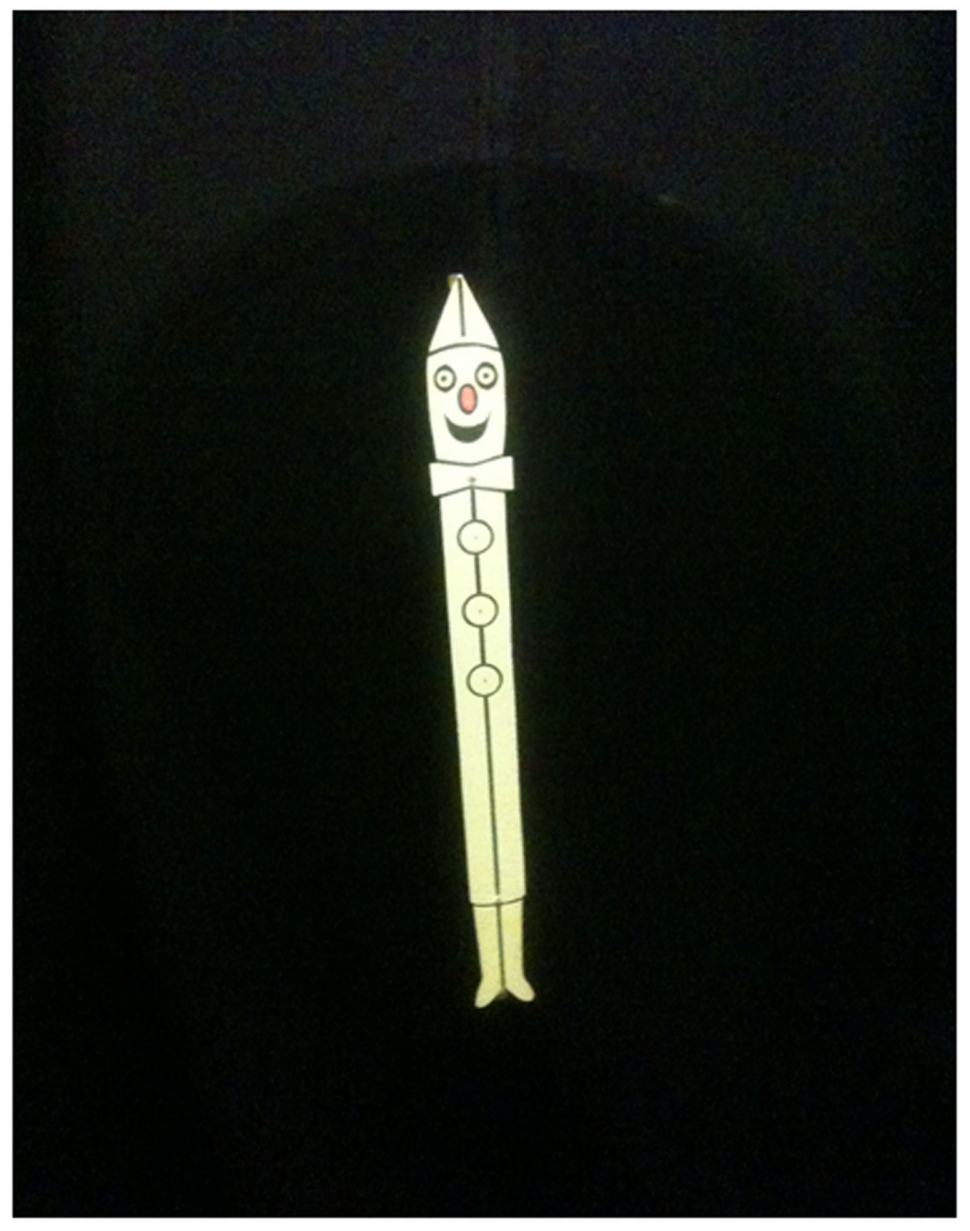
Subjective visual vertical equipment. The fluorescent cardboard looking like a clown used for the vertical perception.

 The test took place in a dark room. Subjects were required to stand upright on the forceplate with their head free to move, their feet placed side by side at an angle of 30° and their heels separated by 4cm. A large dark curtain was suspended from the ceiling to form a semi cylindrical black space around the subject devoid of any visual references. On this curtain, we projected a moving pseudorandom-dots visual pattern creating an optokinetic visual stimulation: 360 dots of 0.34cm (0.235°) diameter subtending 12° (~41.69cm) of the visual field with a density of 2637dots/m². The dots rotated clockwise or counterclockwise with constant angular velocities: 80°/s and 120°/s. The visual vertical perception was assessed with a homemade subjective visual vertical (SVV) system composed of a phosphorescent tube and a fluorescent cardboard looking like a clown (see Figure 1), which could be moved to the left or to the right through the use of a remote control. This clown was placed two meters away from the subject, at eye level. The experimenter could monitor on a screen the degrees of tilt of the clown given by each subject. 

### Experimental procedure

**Figure 2:**
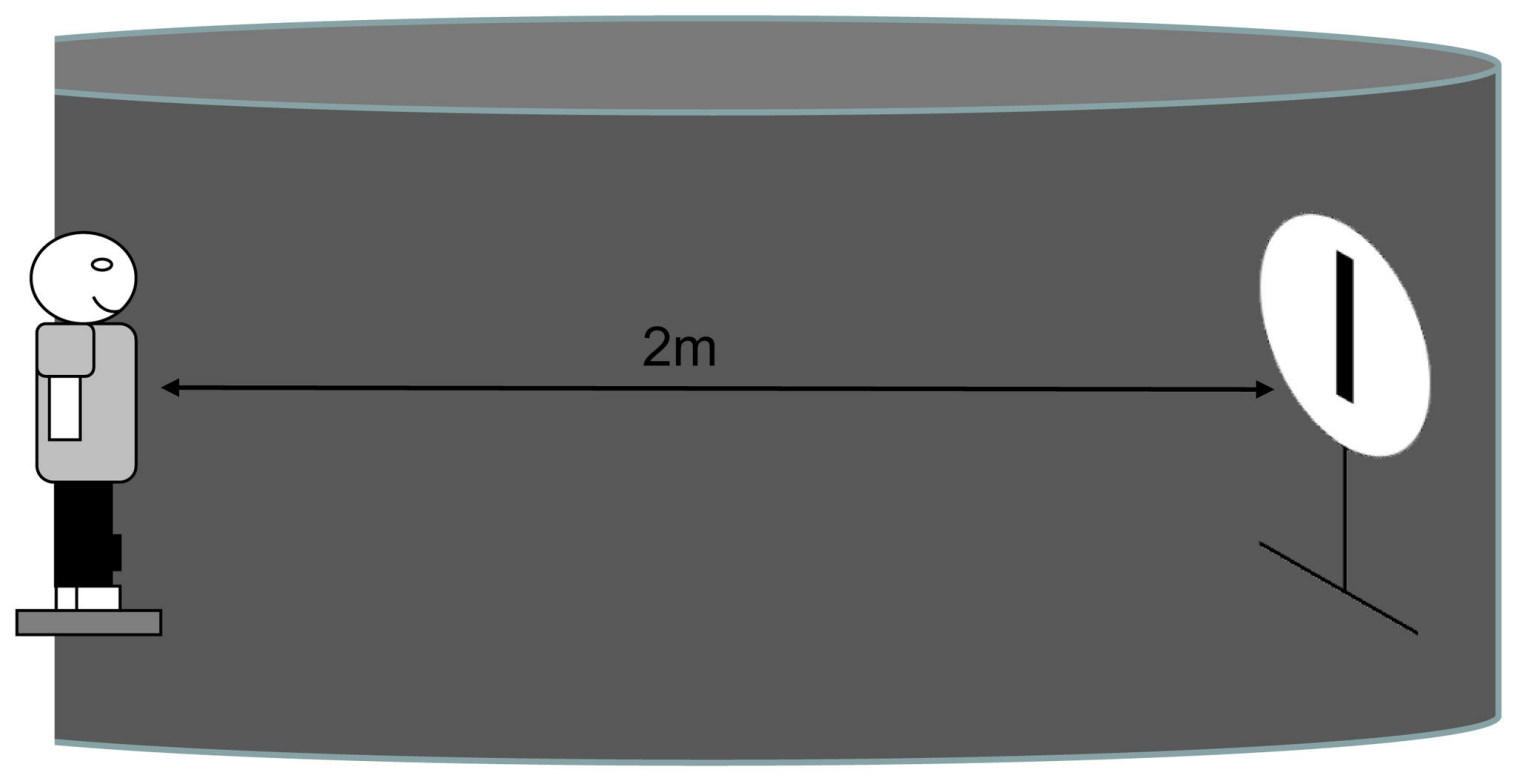
Experimental set-up. The children are on the postural plateform, the visual subjective at 2 meter in front of them, with the black curtain around them in the dark.

Subjects were asked to stand upright on the forceplate in the dark room, arms side by side, breathing normally, not speaking nor clenching their teeth. They held the remote control with their hands in front of their belly button (see Figure 2). The movement required by the control remote was just a contraction of the thumbs, and the keyboard was only 40g. On the keyboard, there was only two buttons: the right button turned the clown to the left, and the left button turned the clown to the right. Each experimental session included 5 conditions to assess postural measure and SVV measure at once. For SVV measurement prior to each trial, the experimenter inclined the clown on the right or on the left side randomly, at different angle of tilt. The subject had then to straighten the clown up until it reaches verticality. At the same time postural stability was recorded.

Four conditions were performed with 4 different optokinetic stimulations presented randomly (conditions OKN+SVV) with visual stimuli velocity respectively at 80°/s left, 80°/s right, 120°/s left, 120°/s right and SVV measurements. For each condition, three trials were run. One more condition was performed in the dark without any optokinetic visual stimuli but with SVV measurement (condition No OKN + SVV).

Two control conditions were also run to assess postural stability: namely one condition (DARK+FIX) where the subject was asked to fixate the clown that was vertically (0°) aligned; and another condition (DARK+EC) where both eyes were closed by a patch while the subject was asked to mentally recall the image of the clown. For each of these conditions two trials were run. 

Before starting the experiment, we trained children on the DARK+SVV condition, without recording it, until we were sure that they understood the task and performed it well. 

### Data acquisition and processing

We recorded the postural stability with a forceplate (principle of stain gauge) produced by Techno Concept (Céreste, France) composed of two dynamometric clogs. The oscillations of the body were measured for 12.8 seconds; the equipment contained an analog to digital converter of 16 bits. The sampling frequency of the CoP was 40 Hz. 

We examined four parameters: the surface of the Center of Pressure, CoP (90% confidence ellipse of CoP area, in mm^2^) which allows to efficiently measure CoP spatial variability [20]; the length of CoP in the medio-lateral axis, which is the path of the CoP in this axis; the standard deviation of the medio-lateral body sway (SdX), which is believed to be controlled by a hip strategy [21]. This parameter was evaluated in order to further investigate the optokinetic effect. We also recorded the mean speed of the CoP that represents a good index of the amount of neuromuscular activity required to regulate postural control [22,23]. Note also that the surface and the length of the CoP are uncorrelated, and the inner surface of the same length may be different [24]. 

Finally, we examined SVV value for each subject by calculating the average of the three trials that were performed for each condition (with optokinetic visual stimuli velocity at 80°/s and 120°/s to the left and to the right respectively, and without any optokinetic visual stimuli). Positive values of SVV indicate a bias to the right side and negative values a bias to the left side. 

### Statistical analysis

 First, a repeated measure ANOVA was done with the velocity and the lateralization of the visual stimuli as main factor (80°/s left, 80°/s right, 120°/s left, 120°/s right) and the adults and children groups as inter-subjects factor, on all postural parameters cited above.

 We then performed an ANOVA using the four aforementioned conditions (OKN+SVV, No OKN+SVV, DARK+FIX, DARK+EC) as main factor and adults and children groups as inter-subjects factor for all postural parameters (Surface of CoP, length of CoP and standard deviation of body sway in medio-lateral axis, and the mean speed of the CoP). An ANOVA test was also performed on the mean SVV values. The five different conditions (80°/s left, 80°/s right, 120°/s left, 120°/s right, Dark) were used as main factor and adults and children groups as inter-subjects factor. 

 The post-hoc comparison was achieved through the Tuckey HSD test; an effect was significant when the *p*-value was below 0.05. 

## Results

### Postural parameters

For the first repeated measure ANOVA test on the lateralization and velocity of the visual stimuli, there were no significant differences between the different visual stimulation (p>0.05). Consequently, all conditions with optokinetic stimulations were averaged, and named OKN+SVV. 

#### Surface of CoP

**Figure 3:**
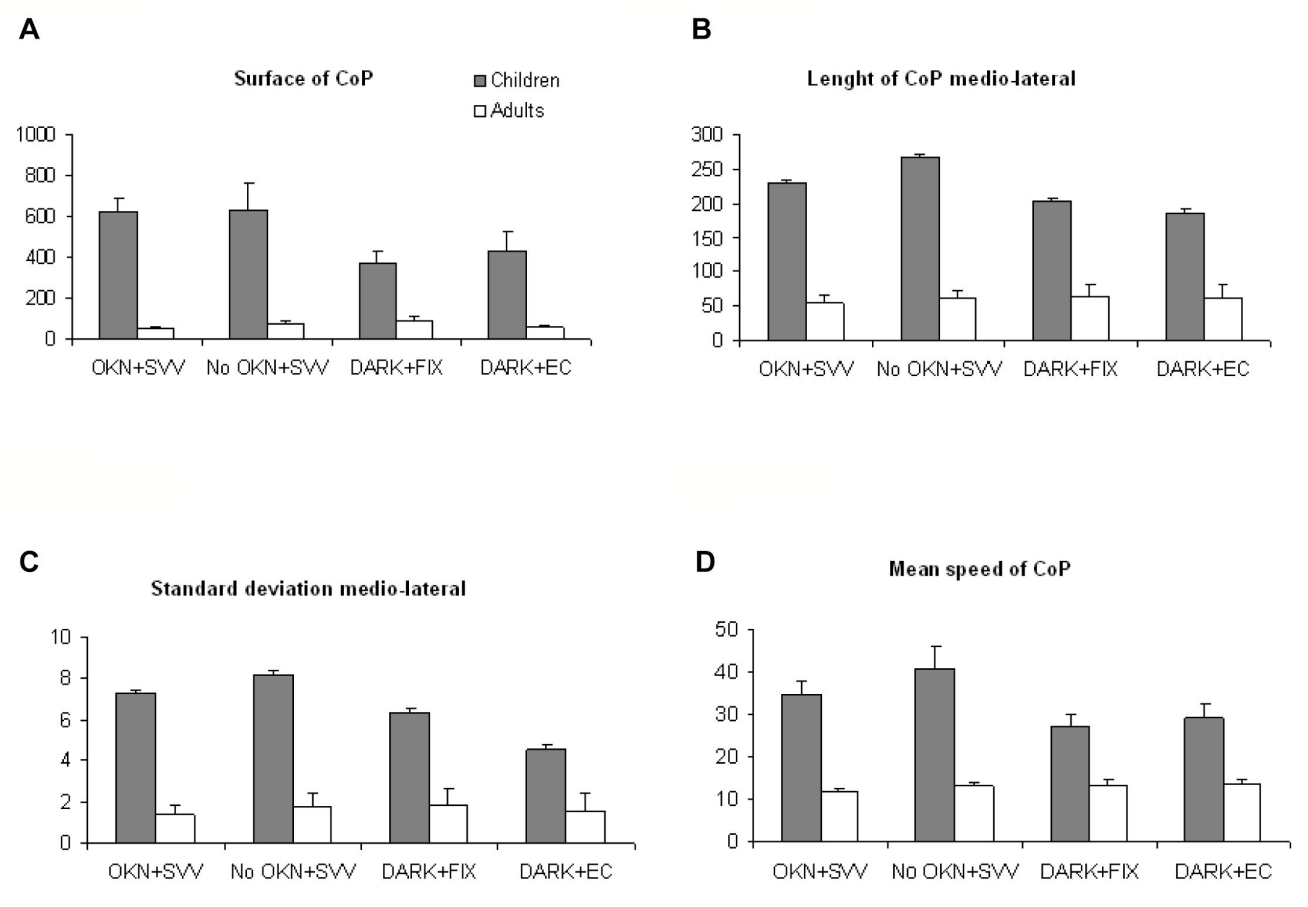
Postural parameters for the different conditions. Mean of Surface of CoP (A), of length of CoP in the medio-lateral axis (B), of standard deviation of medio-lateral body sway (C) and of mean speed of CoP (D) for the four different conditions (OKN+SVV, No OKN+SVV, DARK+FIX and DARK+EC) for adults and children. Verticals bars indicate the standard error.

Figure 3A shows the surface of CoP for the four different conditions respectively for adults and children. The ANOVA test showed a significant effect of group (F_(1,20)_=108.75, p < 0.001), no effect of condition (F_(3,60)_=2.22, p=0.095) and an interaction between condition and groups (F_(3,60)_=2.92, p<0.041). The post-hoc test showed that for all conditions, adults had a statistically significant smaller surface of CoP (mean: 69.16 ± 47.27mm^2^) than children (mean: 512.94 ± 303.78mm^2^). In children only there was a tendency for a smaller CoP surface for the condition DARK+FIX and DARK+EC than for the condition with OKN+SVV and No OKN+SVV, but this tendency did not reach statistical significance. Adults did not show any significative differences with the post-hoc test. 

#### Length of CoP in the medio-lateral axis

Figure 3B shows the length of CoP in the medio-lateral axis for the four different conditions, respectively for adults and children. The ANOVA test showed a significant effect of group (F_(1,20)_=58.01, p< 0.001), a significant effect of condition (F_(3,60)_=5.06, p<0.0034) and an interaction between group and conditions (F_(3,60)_=5.92, p<0.0013). The post-hoc test showed that for all conditions, adults had a statistically significant smaller length of CoP in the medio-lateral axis (mean: 60.44 ± 15.35mm) than children (mean: 221.61 ± 87.49mm). Furthermore, the No OKN +SVV condition showed significantly larger length of CoP in the medio-lateral axis (mean: 154.7 ± 124.43mm) for all subjects compared to the DARK+FIX condition (mean: 127.14 ±89.88mm; p<0.029) and to the DARK+EC condition (mean: 118.33 ± 75.47mm; p<0.0024). Finally, the length of CoP in the medio-lateral axis for children was larger in the No OKN +SVV condition (mean: 267.46 ±100.28mm) compared to the DARK+FIX condition (mean: 202.59 ± 82.97mm), or the DARK+EC condition (mean: 186.11 ± 58.6mm). In children the length of the CoP in the medio-lateral axis appeared to be smaller in condition DARK+FIX and DARK+EC than in condition OKN+SVV and No OKN+SVV, but this was not statistically significant.

#### Standard deviation of medio-lateral body sway

Figure 3C shows the standard deviation of medio-lateral body sway for the four different conditions, for adults and children respectively. The ANOVA test showed a significant effect of group (F_(1,20)_=18.57, p< 0.001), a significant effect of condition (F_(3,60)_=3.83, p<0.014) as well as an interaction between group and condition (F_(3,60)_=3.5, p<0.021). The post-hoc test showed that for all conditions, adults had a statistically significant smaller standard deviation of medio-lateral body sway (mean: 1.63 ± 0.71mm) than children (mean: 6.59 ± 4.59mm). Furthermore, the standard deviation of the medio-lateral body sway in children was statistically larger in NO OKN +SVV condition (mean: 4.68 ± 5.43mm) than in DARK+EC condition (mean: 2.89 ± 2.36mm; p<0.0081). Children had also a smaller standard deviation of medio-lateral body sway in the OKN+SVV condition (mean: 7.32 ± 3.07mm) compared to the No OKN +SVV condition (mean: 8.1 ± 6.57mm), but a larger standard deviation of medio-lateral body sway in the OKN+SVV condition (mean: 7.32 ± 3.07mm) compared to DARK+EC condition (mean: 4.52 ± 2.58mm). In children, there was a tendency for a smaller lateral body sway for the DARK+FIX and DARK+EC conditions than for the OKN+SVV and No OKN+SVV conditions, but this tendency did not reach statistical significance.

#### Mean speed of CoP

Figure 3D shows the mean speed of CoP for the four different conditions, for adults and children respectively. The ANOVA test showed a significant effect of group (F_(1,20)_=48.51, p< 0.001), a significant effect of condition (F_(3,60)_=5.49, p<0.002) and an interaction between group and conditions (F_(3,60)_=6.81, p<0.001). The post-hoc test showed that for all conditions, adults had a statistically significant smaller mean speed of CoP (mean: 12.87 ± 3.14 mm/s) than children (mean: 32.94 ± 12.76 mm/s). Furthermore, it showed that the No OKN+SVV condition (mean: 25.59 ± 17.98mm/s) was associated with significantly higher CoP speed for all subjects compared to the DARK+FIX condition (mean: 19.53 ± 9.43mm/s; p<0.0019) and to the DARK+EC condition (mean: 20.68 ± 10.98 mm/s; p<0.015). Children had also a larger mean speed of CoP in the No OKN+SVV condition (mean: 40.76 ± 16.63mm/s) compared to the FIX condition (mean: 27.06 ± 8.45mm/s), and to EC condition (mean: 29.24 ± 10.91mm/s). There was a slight tendency for a lower mean speed of CoP in DARK+FIX and DARK+EC than in OKN+SVV and No OKN+SVV conditions, but this difference was not statistically significant.

#### Subjective visual vertical measure

**Figure 4:**
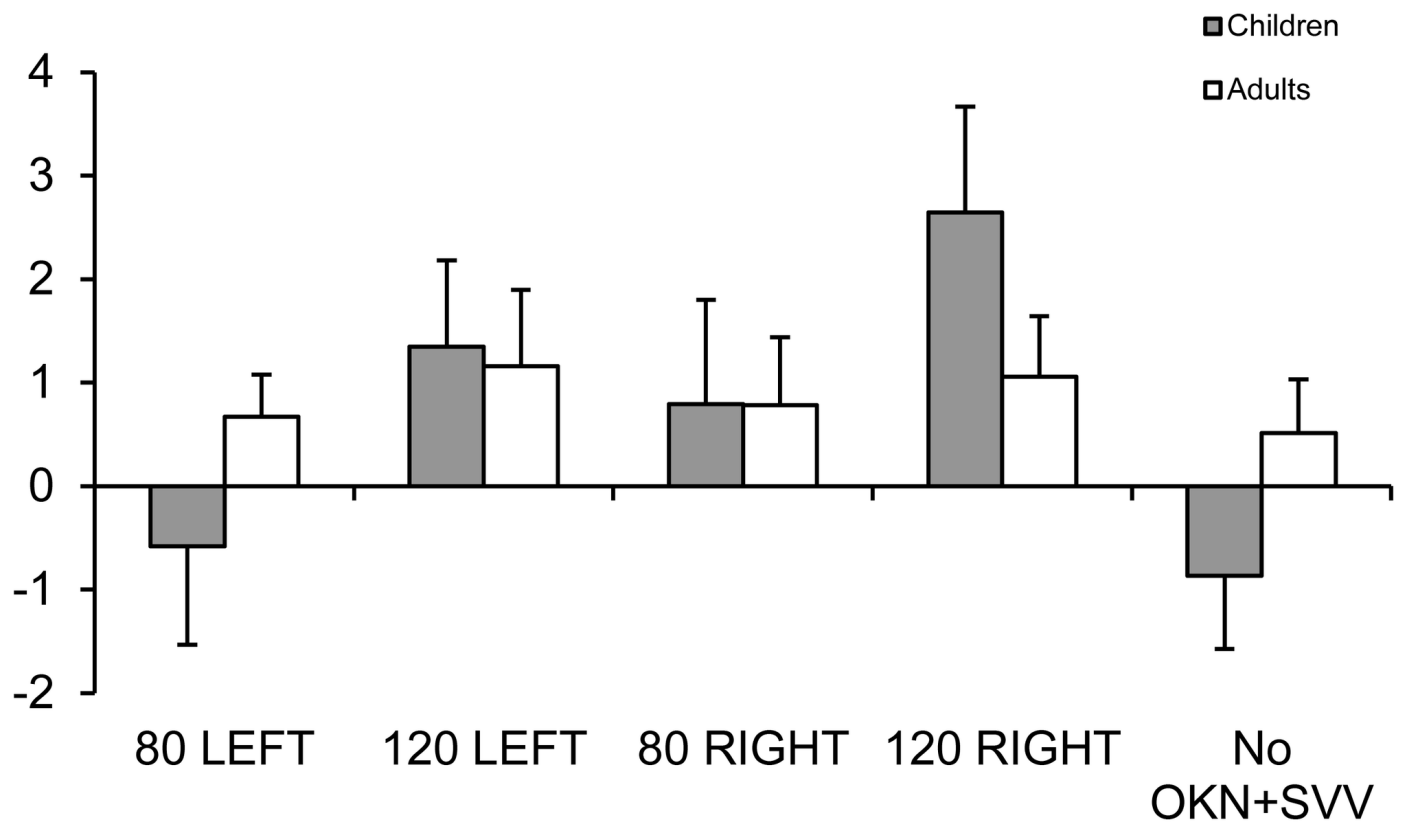
Mean Subjective visual vertical measure. Mean values of the SVV for the five different conditions (OKN+SVV at 80°/s toward the left and the right, at 120°/s toward the left and the right, and the condition No OKN+SVV) for adults and children.

Figure 4 shows SVV mean values for the five different conditions (OKN+SVV at 80°/s toward the left and the right, at 120°/s toward the left and the right, and the condition No OKN+SVV) for adults and children. The ANOVA test showed no significant effect of group (F_(1,20)_=0.045, p=0.83), but a significant effect of condition (F_(3,60)_=4.87, p<0.001). The post-hoc test showed that SVV for all subjects is less variable for OKN+SVV at 80°/s left (mean: 0.10 ± 2.3°) than at 120°/s right (mean: 1.78 ± 2.7°). The SVV was also less variable in the No OKN SVV condition (mean: -0.12 ± 2.08°) than in the OKN+SVV condition at 120°/s right (mean: 1.78 ± 2.7°). The ANOVA test also showed an interaction between group and condition (F_(3,60)_=2.53, p<0.047). The figure shows that the accuracy of the subjective visual vertical in children is less variable for No OKN+SVV and OKN+SVV at 80°/s left conditions than for the other conditions (80°/s right, 120°/s left and 120°/s right). The post-hoc test showed some significant differences only between OKN+SVV at 80°/s left (mean: -0.58 ± 3°) and the OKN+SVV at 120°/s right condition (mean: 2.64 ± 3.24°). Also, the SVV accuracy in the OKN+SVV at 120°/s right condition (mean: 2.64 ± 3.24°) was significatively different from the No OKN+SVV condition (mean: -0.87 ± 2.23°). For each individual case the hand lateralisation did not correspond to the measured SVV bias.

## Discussion

The main findings of this study are as follows: (i) Postural stability is poorer in children than in adults; (ii) in children effects of visual perturbation (OKN stimuli) and visual suppression (dark condition) are observed for all but one parameter (Surface of CoP); (iii) the No OKN+SVV condition is the condition where the instability is higher for all subjects; (iv) SVV values are more variable and less precise in children with respect to adults; furthermore the SVV bias for all adults is to the right while for children the SVV is variable, depending on the condition. All these results will be discussed below. 

### Postural stability children versus adults

Our results showed that children were less stable than adults in all tested conditions (with and without vision stimulation) and for all measured parameters. These results are in line with the existing data already available in the literature, showing that postural stability improves along childhood and adolescence [25–29]. In particular, children younger than ten years are reportedly less efficient in maintaining either static or dynamic balance. Kirshenbaum et al. [30] showed that adult-like balance control strategies begin to appear in 7-8 year-old children, characterized by head–trunk coordination [25]. The children in our study were aged from 6 to 8 years, and it seems that they did not perfect their head-trunk coordination, hence accounting for the fact that children showed larger postural instability in all conditions when compared with adults. Note however that head-trunk movement have not been recorded in this study. 

### Effect of the visual stimulation in children

Our results showed that for all postural parameters (except for the Surface of CoP) there was a significant task effect: the No OKN+SVV condition was the condition where children were the most instable compared to visual fixation or closed eyes, and to the OKN+SVV condition, but only for the standard deviation of medio-lateral body sway. The paradigm used in the present study is a dual-task since subject had to control posture and simultaneously perceive and appreciate subjective visual vertical, which is a cognitive task. Studies on the effect of a dual-task on postural control showed a deterioration of the postural stability in children and adolescent due to the fact that children shift their attention toward the secondary task leading to worse postural control [28,29,31]. Comparing children and adults, these authors proposed that the attentional resources of children, unlike those of adults, might be reduced so as to unable them to properly allocate enough attention for each task (postural and secondary) or limit children in processing all information. These hypotheses are in line with the U-shaped non-linear interaction model of Lacour et al. [32] suggesting that body sway increased when subjects accomplished a highly attentional demanding secondary task. In the present study, the level of attention called by the subjective visual vertical task was quite high leading to a deterioration of the postural control in children. We also know that children are more visuo-dependent than adults for their postural control [25,28,29] and in our experiment, children were in total darkness. They did not have any visual spatial references to help them stabilize their body and control their posture. This may explain why the No OKN+SVV condition was leading to more instability for children. For all postural parameters the data showed a tendency for being less instable in the DARK+FIX and DARK+EC condition than in SVV conditions. DARK+FIX and DARK+EC conditions, used typically for posture measurement as control condition, are performed while the subject is required to fixate or imagine a small target (usually a dot or a small picture). In our study, the target was a vertical bar, which probably gave an indication for verticality and may have helped the subjects to stabilize their posture. 

Finally, it should be noted that for the standard deviation of medio-lateral body sway, the results revealed that in the OKN+SVV condition children were more instable than in the DARK+EC condition. This result may be due to the fact that the optokinetic condition was performed/achieved through visual stimulation acting in the same plan (medio-lateral), thus affecting the medio-lateral body sway. 

Surprisingly, there was no difference in postural stability between the OKN + SVV and No OKN + SVV conditions. We suspect that it could be due to the OKN stimuli in our set up, which provide a lighting source able to reveal some subtle spatial references about the environment used by the child as spatial complementary references. 

### Vertical visual subjective values

As predicted, the values of subjective visual vertical for children were more variable and less accurate than those reported in adult population. The lack of visual reference or the modified visual reference (optokinetic stimulation) might alter the allocentric reference frame (based on visual information) thus perturbing the perception of the verticality. This may account for higher values in children. Children are more dependent on visual information and their accuracy of verticality at this age (6-8 years old) is not high yet. Furthermore, children showed also more variability values of the SVV bias to the left (negative bias) and to the right (positive bias) while adults only had a bias to the right. This could not be explained by the laterality of the subjects as only two adults and three children were left handed (the other being all right handed) in our groups. May be children showed more variability in their estimation of the subjective visual vertical because they had less experience in weighting the different sensory information, and thus showed more of the so-called inter-subject variability as suggested by Isableu et al. [14]. It is possible that the cortical maturation of 6 to 8 years old children does not permit a precise enough analysis of each of the multisensory referent information to construct their visual vertical. This hypothesis is in line with the recent research of Lopez et al. [33] exploring the subjective visual vertical in adult subjects with EEG recordings. They showed that SVV involves large cortical processing with an early activation of the temporal-occipital cortical areas (75-105 ms) and a later activation of the parietal-occipital cortical areas (260-290 ms). The first early activation would involve a ventral visual pathway relative to the attentional treatment of perception and orientation, while the later activation could involve mutlisensorial integration of gravity and of the body position using vestibular, muscular and proprioceptive informations. It is highly probable that these central processing did not reach complete maturation in our children group. On the other hand, children may not have been able to memorize all the multisensory reference frames available by lack of experience. The effect of professional experience or training on the variability and precision of the SVV evaluation supports this hypothesis [34,35]. 

Our data showed that adults presented a right bias of their subjective vertical in OKN condition, as well as children in most condition. These results surprised us because it was not in line with results already published in adults by Lopez et al. [36] where the same optokinetic stimulation was used. In their study all patients presented a deviation of the subjective vertical in the direction of the rotation of the optokinetic stimulation. We think that this bias in our study might come from some subtle spatial references about the environment due to the lighting source of the OKN stimuli. These parts of our results concerning the effect of optokinetic stimulation on the subjective vertical need to be confirmed with an improved set up in order to completely exclude spatial references during OKN stimuli. 

Further studies measuring vertical visual subjective on a broader age range population of children is planned to characterise the development of SVV perception along childhood. 

## Conclusion

Our study shows that children from 6 to 8 years of age are more instable and have a less precise evaluation of the verticality than young adults. This is probably due to the maturation of the cortical processes involved in the perception of verticality that has not been achieved yet, and also to a limitation of attention. Central nervous system development and training contribute to the achievement of vertical evaluation and postural stability. Although, further studies are needed on a larger population of children with a broader age range to study the development of the vertical perception. 
